# The Difference in Prognostic Factors between Early Recurrence and Late Recurrence in Estrogen Receptor-Positive Breast Cancer: Nodal Stage Differently Impacts Early and Late Recurrence

**DOI:** 10.1371/journal.pone.0063510

**Published:** 2013-05-22

**Authors:** Sung Gwe Ahn, Hak Min Lee, Sang-Hoon Cho, Suk Jin Bae, Seung Ah Lee, Seung Hyun Hwang, Joon Jeong, Hy-De Lee

**Affiliations:** 1 Department of Surgery, Yonsei University Medical College, Seoul, Republic of Korea; 2 Department of Statistics and Actuarial Science, Soongsil University, Seoul, Republic of Korea; Health Canada, Canada

## Abstract

**Background:**

Probability of recurrence in patients with estrogen receptor (ER)-positive breast cancer remains constant for long periods. We compared tumor burden impact on late versus early recurrence in our cohort with long-term follow-up.

**Methods:**

Five hundred and ninety five patients diagnosed with ER-positive breast cancer between 1989 and 2001 were classified into three groups: early recurrence within 5 years, late recurrence after 5 years, and no recurrence. We identified prognostic factors among the groups using logistic regression analysis.

**Results:**

At median follow-up of 11.7 years, among 595 ER-positive women, 98 (16.4%) had early recurrence and 58 (9.7%) had late recurrence. On multivariate analysis, higher nodal stage (N0 vs. N2, odds ratio [OR] 3.189; N0 vs. N3, OR 9.948), higher histologic grade (grade 1 vs. grade 2, OR 3.896; grade 1 vs. grade 3, OR 5.945), age >35 years (OR 0.295), and receiving endocrine therapy (OR 0.293) affected early recurrence. Compared to no recurrence, receiving endocrine therapy (OR 0.285) was solely related to decreased risk of late recurrence. Increased risk of early recurrence was noted with the higher nodal stage when early and no recurrences were compared. This phenomenon was not found in late recurrence. In the last comparison between the early and late recurrence, higher nodal stage (N0 vs. N3, OR 16.779) and higher histologic grade (grade 1 vs. grade 3, OR 18.111) repeatedly weighted for early recurrence.

**Conclusions:**

Nodal burden had an attenuated influence on late recurrence, which suggests that, unlike early recurrence, tumor biology might have a more important role than tumor load for late recurrence in ER-positive disease.

## Introduction

Breast cancer is the most common cancer in women, with approximately 1.5 million new cases diagnosed annually worldwide, a lifetime risk of up to 12%, and a risk of death of up to 5% in Western countries [1]. With advances in early detection and improvements in breast cancer treatment, markedly increasing long-term survivors who remain at risk of recurrence are raising issues for oncologists [2]–[4]. Therefore, the identification of factors influencing late recurrence after 5 years has become increasingly important.

Previous studies reported that the risk of early relapse is greater for women with estrogen receptor (ER)-negative than ER-positive breast cancer, but late relapses are more common in ER-positive than ER-negative disease [4]–[6]. Although the use of endocrine therapy in clinical practice remarkably enhanced survival outcomes of ER-positive patients [7], the probability of recurrence among patients with ER-positive disease remains constant over time [4]–[6]. In this context, recent studies have focused on the residual risk of late recurrence among long-term survivors with ER-positive disease [8], [9].

Tumor size and number of involved lymph nodes representing tumor burden are the most important prognostic factors for breast cancer recurrence [10], [11]. Tumor relapse in the early period following treatment has conventionally been considered a problem of excess tumor burden regardless of ER status. Previous studies reported that nodal stage was associated with early recurrence within 5 years in ER-positive breast cancer [12], [13], and this conventional concept was also confirmed in patients with ER-negative disease. With respect to late recurrence after 5 years, several studies demonstrated that advanced stage of the primary tumor raised the risk of late relapse [11], [14], [15], but these studies also included ER-negative breast cancer patients.

With this landscape, we hypothesized that tumor load, recognized as an important prognostic factor for early recurrence, might influence late recurrence differently in ER-positive disease. In this study, we investigated the prognostic factors of early recurrence within 5 years and late recurrence after 5 years in ER-positive breast cancer patients.

## Patients and Methods

### Patients

The institutional review board (IRB) of Gangnam Severance Hospital, Yonsei University, Seoul, Korea, approved the study in accordance with good clinical practice guidelines and the Declaration of Helsinki (local IRB approval number: 2012-0199). The need for informed consent was waived because of the retrospective design. Patients included in this study were retrospectively selected from the database of breast cancer patients treated between January 1991 and December 2001 at Gangnam Severance Hospital, Yonsei University Medical College, Seoul, Korea. During the period, 1,329 patients were treated for breast cancer and entered into the database. The follow-up protocol included planned regular visits every six months and requests for missed appointments with telephone calls were made to minimize patient loss and raise the accuracy of survival data. The last update of the clinical database was in April 2012. Among 1106 patients with known ER status, 677 had ER-positive breast cancer. Patients with ductal carcinoma in situ, distant metastases at initial assessment, and clinical or pathological stage T4 disease were excluded. Those who received preoperative chemotherapy were eliminated from this study because of inaccurate assessment for clinical tumor staging. Patients with unavailable information of tumor characteristics and endocrine therapy were also excluded from these analyses. Contralateral breast cancer was not defined as recurrence because it could not be determined whether the disease was true recurrence or new primary. Similarly, ipsilateral breast tumor recurrence following breast-conserving surgery could not be determined as recurrence because there was a lack of pathological information. As a result, the patients with metachronous contralateral breast cancer and ipsilateral breast tumor recurrence were excluded.

Following the exclusion, we retrieved data from 595 consecutive patients with ER-positive breast cancer who underwent curative surgery. Before February 1999, ER status was determined by the ligand binding assay, and tumors were considered ER-positive if they scored>10 fmol/mg [16]. From February 1999, the immunohistochemical (IHC) method for ER staining was introduced and replaced the biochemical method. T and N stage were classified according to the American Joint Committee on Cancer (AJCC), 6th edition. The modified Scarf-Bloom-Richardson grading system was used for tumor grading.

The site of recurrence was classified as local (ipsilateral breast or chest wall), regional (ipsilateral axillary, infraclavicular, internal mammary, or supraclavicular), or distant metastasis (any other site). Early recurrence was defined as initial recurrence within 5 years following curative surgery irrespective of site. Likewise, late recurrence was defined as initial recurrence after 5 years. According to the timing of first the recurrence, all patients were stratified into three groups (early recurrence, late recurrence, and no recurrence during the follow-up period). Receiving endocrine therapy was defined as a patient who completed a 5-year course of endocrine therapy. The patients underwent recurrence during the period of endocrine therapy were also classified as the group of endocrine treatment. Patients receiving endocrine therapy after first relapse were excluded from the group treated with endocrine agents. Patients receiving adjuvant radiation therapy, which affects recurrence rates, were also investigated.

### Statistics

The Chi-square test was used to compare baseline tumor characteristics. The primary end-point was recurrence-free survival defined as the interval from curative surgery to the first recurrence of breast cancer irrespective of the site. The binary logistic regression models were employed to compare characteristics between the no recurrence and early or late recurrence groups. The significant prognostic factors associated with early or late recurrence were selected according to the Akaike Information Criterion in a stepwise fashion while avoiding over-parameterization.

The secondary end-point was distant metastasis-free survival that has a major impact on overall survival. Multivariate comparison was used to identify prognostic factors significantly associated with early or late metastasis. The number of recurrence sites was greater than the number of patients with recurrence because of the patients with multiple recurrent sites including both loco-regional recurrence and distant metastasis.

For examination a trend between recurrence groups and nodal stage, linear-by-linear association chi-squared test was performed. All statistical analyses were performed using the SPSS statistics program version 18.0 (SPSS Inc., Chicago, IL) and R (http://www.r-project.org) software. A *p*-value<0.05 was considered statistically significant.

## Results

### Recurrence Pattern

In 595 ER-positive patients, the median age at diagnosis of primary tumor was 45 years (range, 23 to 80). At a median follow-up of 11.7 years (range, 0.3 to 20.4), 156 recurrences occurred, with 98 early recurrences and 58 late recurrences reported. There were 91 early and 58 late distant metastasis reported, leaving seven patients who had sole loco-regional recurrence without distant metastasis. According to the site of recurrence, there were 26 local, seven regional, and 149 distant recurrences. During the follow-up period, 119 deaths occurred.

### Surgical, Endocrine, and Radiation Treatments

There were 393 (66.1%) patients who underwent mastectomy and 202 (33.9%) received breast-conserving surgery. Among 502 patients receiving endocrine treatment, 275 women were treated with tamoxifen, 192 with toremifen, and one with letrozole. Eighteen patients switched from tamoxifen to toremifen or toremifen to tamoxifen and 16 had extension with letrozole after completing 5 years of tamoxifen treatment. Among the patients receiving endocrine therapy, 107 had a recurrence with 67 occurring during the treatment period. Of these, 48 continued the treatment or changed endocrine agents while 19 stopped endocrine therapy. Forty patients had a recurrence after completing 5 years of treatment.

263 patients received adjuvant radiation therapy (44.2%). In 202 patients with breast conservation surgery, radiation therapy was performed for 190 patients (94.1%). Among 393 patients with mastectomy, 73 women received adjuvant radiotherapy (18.6%).

### Patient Characteristics

Baseline tumor characteristics are listed and compared in the paired groups (Table 1). Compared with the late recurrence group, the early recurrence group tended to have a younger age, and higher nodal stage and histologic grade. A similar trend was found that women in the early recurrence group had a younger age, larger tumor size, higher nodal stage, higher histologic grade, more lymphovascular invasion, and received endocrine therapy with a lower frequency compared to the no recurrence group. Compared with women without recurrence, women with late recurrence had larger tumor size and higher nodal stage. Moreover, the patients not receiving endocrine therapy tended to have more frequent late recurrence than the patients who received endocrine therapy.

**Table 1:** Patient characteristics according to recurrence pattern.

	No. patients			χ^2^ test			
Characteristics	Early recurrencewithin 5 years:Group 1 (n = 98)	Late recurrenceafter 5 years:Group 2 (n = 58)	No recurrence:Group 3 (n = 439)	Group 1 vs.Group 2	Group 2 vs. Group 3		Group 3 vs.Group 1
**Age**				0.022	0.410	<0.001	
Age<35 (n = 63)	25	6	32				
Age≥35 (n = 532)	73	52	407				
**Tumor size**				0.848	0.006	<0.001	
≥2 cm (n = 236)	74	15	197				
<2 cm (n = 359)	24	43	242				
**N stage**				0.014	0.001	<0.001	
N0 (n = 325)	28	21	276				
N 1 (n = 172)	26	24	122				
N2 (n = 55)	18	9	28				
N3 (n = 43)	26	4	13				
**Histologic grade** a				0.002	0.101	<0.001	
1 (n = 163)	13	19	131				
2 (n = 249)	50	32	167				
3 (n = 77)	21	3	53				
**Progesterone receptor** a				0.541	0.430	0.053	
Presence (n = 463)	73	48	342				
Absence (n = 74)	18	9	47				
**Lymphovascular invasion** a				0.267	0.083	<0.001	
Presence (n = 33)	13	4	16				
Absence (n = 412)	52	36	324				
**Endocrine therapy**				0.938	<0.001	<0.001	
Yes (n = 502)	67	40	395				
No (n = 93)	31	18	44				
**Surgical method**				0.582	0.702	0.203	
Mastectomy (n = 393)	70	39	284				
BCS (n = 202)	28	19	155				
**Radiotherapy**				0.945	0.674	0.433	
Yes (n = 263)	40	24	199				
No (n = 332)	58	34	240				

Abbreviation: BCS, breast-conserving surgery.

a Values with missing data.

Regarding the rates of radiation therapy, there was no difference according to recurrence status. In this study, radiation treatment did not affect the pattern of recurrence in ER-positive patients.

### Prognostic Factors using Binary Logistic Regression Model

In comparison to no recurrence, age under 35 years (odds ratio [OR] 0.295, 95% CI 0.120–0.730), higher nodal stage (N0 to N2, OR 3.189, 95% CI 1.210–8.410; N0 to N3, OR 9.948, 95% CI 3.828–25.853), higher histologic grade (grade 1 to grade 2, OR 3.896, 95% CI 1.365–11.124; grade 1 to grade 3, OR 5.945, 95% CI 1.892–18.685), and receipt of endocrine therapy (OR 0.293, 95% CI 0.130–0.659) were significant prognostic factors for early recurrence within 5 years (Table 2). With respect to no metastasis, higher nodal stage (N0 to N3, OR 11.349, 95% CI 4.166–30.922), higher histologic grade (grade 1 to grade 2, OR 3.285, 95% CI 1.140–9.464; grade 1 to grade 3, OR 4.503, 95% CI 1.409–14.395), and receipt of endocrine therapy (OR 0.198, 95% CI 0.087–0.453) were significantly associated with an early metastasis within 5 years compared to no metastasis (Table 2). In both comparisons, higher nodal stage was significantly associated with an increased risk of early recurrence or metastasis.

**Table 2:** Binary logistic regression analysis comparing recurrence or metastasis within 5 years of diagnosis with no recurrence or no metastasis.

	Any type of recurrence			Distant metastasis		
Characteristics	No. patients	OR (95% CI)	P value	No. of patients	OR (95% CI)	P value
**Age**			0.008	n/a^b^	n/a^b^	n/a^b^
Age<35	31	Reference				
Age> = 35	315	0.295 (0.120–0.730)				
**Tumor size**	n/a^b^	n/a^b^	n/a^b^			0.149
≥2 cm				141	Reference	
<2 cm				204	1.852 (0.803–4.273)	
**N stage**			<0.001			<0.001
N0	178	Reference		179	Reference	
N1	107	1.595 (0.719–3.537)	0.250	106	1.513 (0.654–3.504)	0.333
N2	32	3.189 (1.210–8.410)	0.019	31	2.573 (0.898–7.388)	0.078
N3	29	9.948 (3.828–25.853)	<0.001	29	11.349 (4.166–30.922)	<0.001
**Histologic grade** a			0.009			0.036
1	108	Reference		108	Reference	
2	176	3.896 (1.365–11.124)	0.011	176	3.285 (1.140–9.464)	0.028
3	62	5.945 (1.892–18.685)	0.002	61	4.503 (1.409–14.395)	0.011
**Endocrine therapy**			0.003			<0.001
No	39	Reference		38	Reference	
Yes	307	0.293 (0.130–0.659)		307	0.198 (0.087–0.453)	

Abbreviation: n/a, not applicable.

a Values with missing data.

b A binary logistic regression model was selected using Akaike Information Criteria (AIC) in stepwise selection. Odds ratios are adjusted for all of the factors listed in the table.

Next, comparison of late recurrence after 5 years and no recurrence suggested that only receipt of endocrine therapy (OR 0.285, 95% CI 0.115–0.704) was related with a decreased risk of late recurrence after 5 years (Table 3). Receipt of endocrine therapy also remained a significant prognostic factor (OR 0.265, 95% CI 0.105–0.666) for metastasis after 5 years compared to no metastasis (Table 3). The negative impact of nodal stage on early recurrence observed in the comparison above was not found in this analysis.

**Table 3:** Binary logistic regression analysis comparing recurrence or metastasis after 5 years of diagnosis with no recurrence or no metastasis.

	Any type of recurrence			Distant metastasis		
Characteristics	No. patients	OR (95% CI)	P value	No. of patients	OR (95% CI)	P value
**Tumor size**	n/a^b^	n/a^b^	n/a^b^			0.156
≥2 cm				141	Reference	
<2 cm				188	1.803 (0.798–4.073)	
**N stage**			0.078			0.074
N0	175	Reference		176	Reference	
N1	112	2.693 (1.256–5.773)	0.011	112	2.817 (1.251–6.344)	0.012
N2	26	2.298 (0.656–8.055)	0.194	28	2.769 (0.819–9.368)	0.101
N3	13	1.265 (0.147–10.926)	0.831	13	1.177 (0.133–10.427)	0.883
**Histologic grade** a			0.064			0.122
1	113	Reference		112	Reference	
2	167	1.837 (0.824–4.096)	0.136	170	1.766 (0.758–4.112)	0.187
3	46	0.388 (0.078–1.932)	0.248	47	0.552 (0.134–2.275)	0.411
**Endocrine therapy**			0.007			0.005
No	32	Reference		31	Reference	
Yes	294	0.285 (0.115–0.704)		298	0.265 (0.105–0.666)	

Abbreviation: n/a, not applicable.

a Values with missing data.

b A binary logistic regression model was selected using AIC in stepwise selection. Odds ratios are adjusted for all of the factors listed in the table.

**Figure 1:**
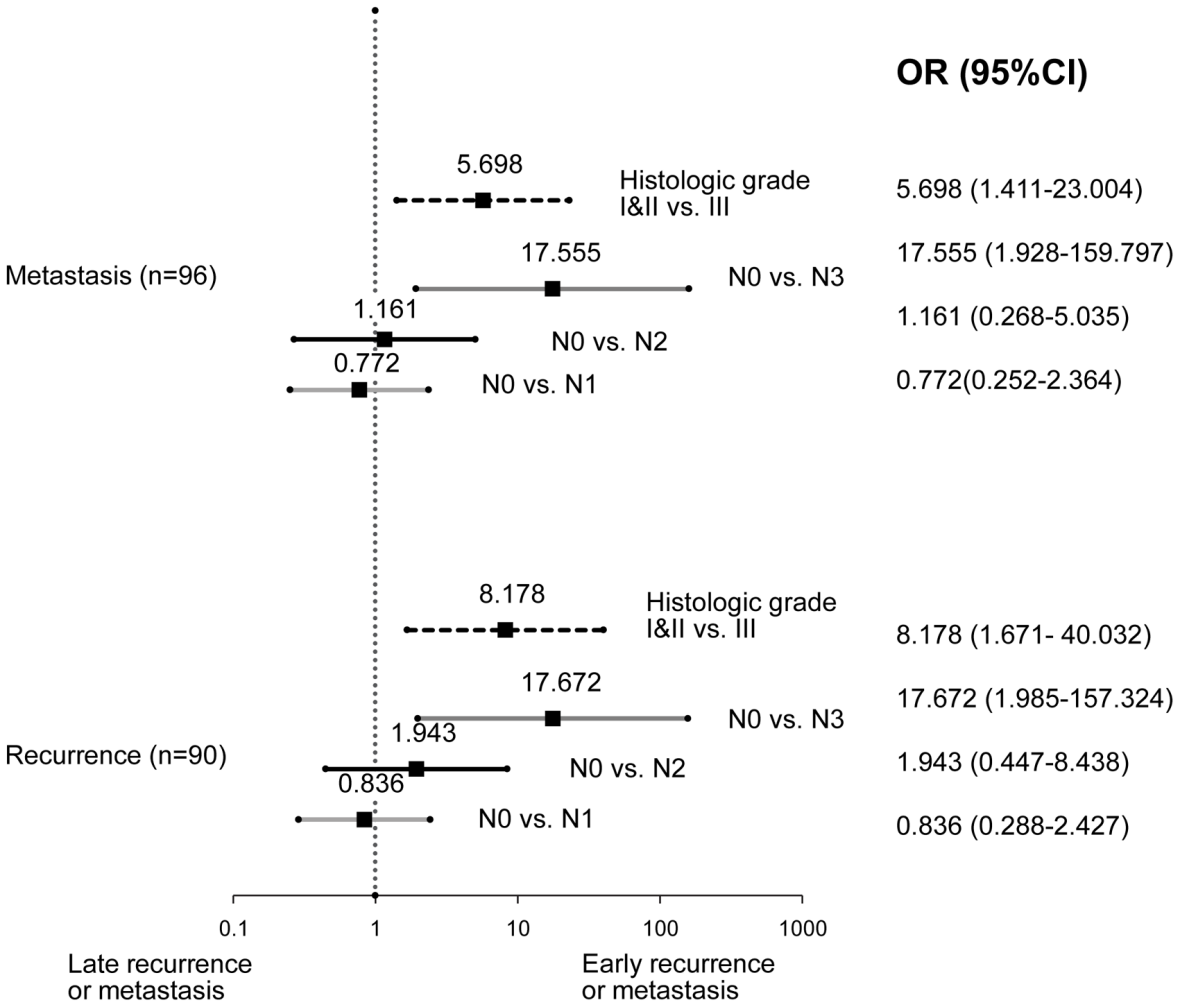
Illustrated odds ratio in the comparison between early recurrence or metastasis within 5 years and late recurrence or metastasis after 5 years. A binary logistic regression model was selected using Akaike Information Criteria in stepwise selection. Odds ratios are adjusted for all of the factors illustrated in the figure.

These analyses identified different prognostic factors for early and late recurrence. In the next step, we directly compared the prognostic factors between early and late recurrence. In the analysis, higher nodal stage (N0 to N3, OR 16.799, 95% CI 1.848–152.353) and higher histologic grade (grade 1 to grade 3, OR 18.111, 95% CI 2.644–124.085) remained as significant risk factors for early recurrence within 5 years (Fig. 1). Comparing early and late metastasis, these two characteristics repeatedly weighted for early metastasis within 5 years (N0 to N3, OR 16.570, 95% CI 1.798–152.670; grade 1 to grade 3, OR 10.783, 95% CI 1.818–63.967; Fig. 1).

The goodness of fit for the binary logistic models employed in this study was investigated using the Hosmer and Lemeshow test and receiver operating characteristic curves. As summarized in Figs. S1, S2, S3, S4, S5, and S6 and Table S1, the goodness of fit tests supported all multivariate comparison models with *P*-values greater than 0.069 and are under the curve values ranged from 0.70 to 0.83.

### Impact of Nodal Stage on Recurrence

**Figure 2:**
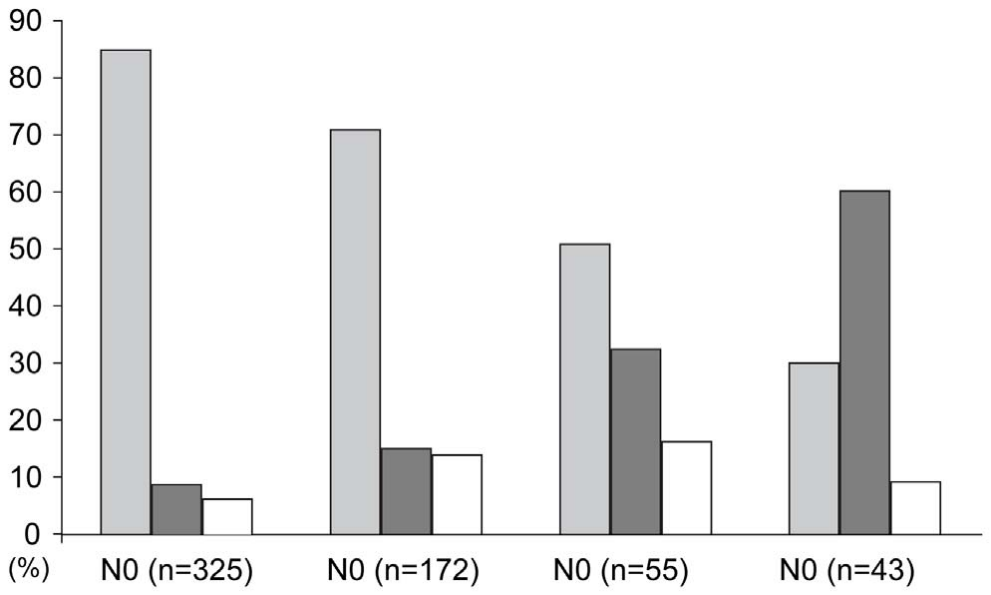
Histograms of three groups based on recurrence: early recurrence within 5 years, late recurrence after 5 years, and no recurrence. Each histogram indicates the proportion of patients with the recurrence groups in each nodal stage. Linear-by-linear association chi-squared test was performed for examination a trend between recurrence groups and nodal stage (*P*<0.001).

We conducted additional investigations to obtain further insight regarding the relationship of nodal stage and recurrence (Fig. 2). In the figure, each histogram indicates the proportion of patients with the recurrence groups in each nodal stage. The proportion of patients with early recurrence was increased in higher nodal stage; however, an inverse trend was observed in the relationship of no recurrence and higher nodal stage. In the pattern with late recurrence, linear trend based on nodal stage was not found.

**Figure 3:**
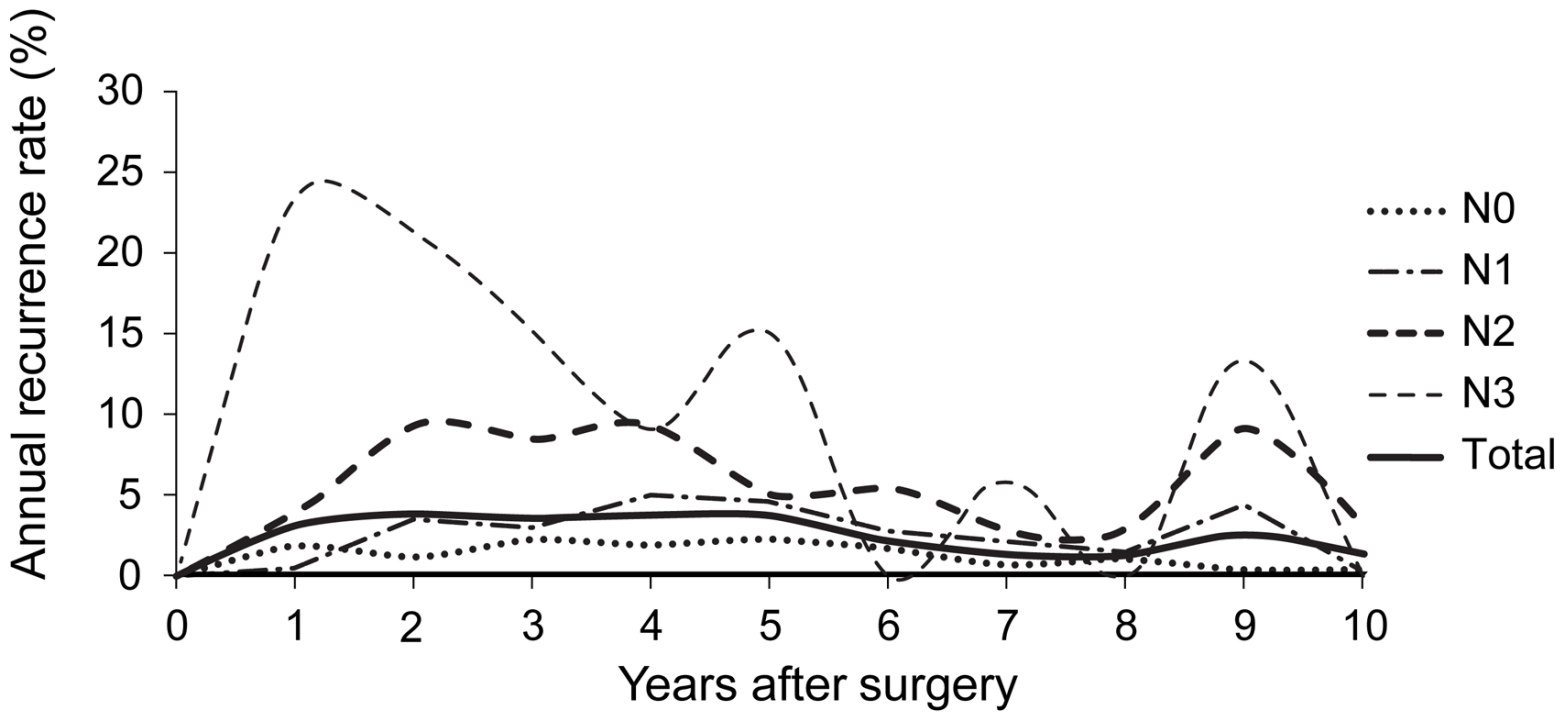
Annual recurrence rate by nodal stage.

Annual recurrence rates by nodal stage during the first 10 years are illustrated in Fig. 3. Overall recurrence ranged from 1.3 to 3.8% and it was 2.6% in the ninth year. The highest recurrence rate was 23.3% in the first year in patients with N3 disease, and it remained high over the first 5 years, then decreased gradually thereafter. Women with N2 stage disease showed a relatively high recurrence rate during years 2 to 4, and the rate slightly decreased after 5 years. The patients with N0 and N1 generally showed low recurrence rate during 10 years (N1, 0.3 to 5.0%; N0, 0.5 to 2.3%).

## Discussion

In a cohort study of ER-positive breast cancer patients with long-term follow-up, the well-known phenomenon of late recurrence was observed in our data. The overall recurrence rate after 5 years persisted constantly (range, 1.3 to 2.6%; Fig. 3). In the comparisons of characteristics among the groups, early recurrence or metastasis group showed remarkable features related to aggressive tumor behavior, such as young age, large tumor size, higher nodal stage, and higher histologic grade (Table 2). The observation that women with these characteristics have a high risk of early recurrence was reported in multiple earlier studies [12], [13]. In contrast, women with late recurrence did not seem to have aggressive tumor characteristics compared with women without recurrence (Table 3). Among tumor characteristics reflecting tumor load, only nodal stage was demonstrated as a prognostic factor associated with early recurrence. Tumor size reported as an important prognostic factor for recurrence in the previous studies was not significantly demonstrated in this study [10]–[13].

In this investigation, we found a different prognostic impact of nodal stage between early and late recurrence in ER-positive women. For early recurrence within 5 years, higher nodal stage linearly increased the hazard of recurrence (N0 to N1, OR 1.595; N0 to N2, OR 3.189; N0 to N3, OR 9.948; Table 2). This linear correlation was not found in the relationship of late recurrence and nodal stage. Of course, women with N1 disease had an OR of 2.693 compared with women with N0 (*P = *0.011); however, the increasing hazard for late recurrence according to higher nodal stage was not observed (N0 to N2, OR 2.298; N0 to N3, OR 1.265, respectively; Table 3). In the final logistic analysis, we compared the two recurrence groups to evaluate the different impact of prognostic factors. Higher nodal stage emerged as a significant risk factor for early recurrence in this final analysis. This prominently decreased effect of nodal stage was similarly observed in the comparison of early metastasis within 5 years and late metastasis after 5 years. The finding of diminished influence of nodal stage on late recurrence compared with early recurrence strongly suggests that factors related to tumor biology have a more pivotal role than tumor load in late recurrence of ER-positive disease. This perspective is concordant with the studies to elucidate tumor biology contributing to late recurrence [17]–[19]. Towards this goal, recent research also identified novel molecules which participate in the process of late relapse [20]–[22] and the classical hypothesis of tumor dormancy was developed to explain late recurrence [2], [11].

However, in parallel comparison between early recurrence and late recurrence, the major impact of nodal stage should be considered. Recently, many investigators sought to distinguish patients who are at risk for early relapse versus late relapse in ER-positive breast cancer using biomarkers or molecular approaches [23]–[25]. These novel methods provided important information in understanding the tumor biology associated with early and late recurrence of ER-positive disease. However, our results suggested that the profound influence of nodal stage on early recurrence should not be overlooked. Prior to parallel analysis of early recurrence and late recurrence, adjustment for the effect of nodal stage is necessary.

In a previous study [9], Kennecke et al. already reported that T and N stages predicted late relapse and death from estrogen responsive early breast cancer in postmenopausal women. In contrast to our study, the authors compared two groups stratified by cohorts without and with late recurrence. Therefore, the different prognostic impact of nodal stage on early vs. late recurrence in ER-positive disease was not explored.

To suppress recurrence, endocrine therapy has been an integral part of adjuvant treatment modalities in ER-positive patients. The endocrine agents provided a great survival benefit for women with ER-positive breast cancer, and selective estrogen receptor modulators and/or aromatase inhibitors showed a significant reduction in recurrence after treatment completion [26], [27]. The protective carryover effect of endocrine therapy was similarly noted in this analysis. Among patients treated with tamoxifen or toremifen, the observed therapeutic effect in the late period was consistent with that of large studies [26], [27]. However, 40 women in this study experienced late recurrence after 5 years despite endocrine therapy. Late recurrence despite endocrine therapy has been a challenging obstacle to overcome.

Retrospective design is a major limitation in this study. This study could be affected by the bias associated with the decision of endocrine therapy or the inherent selection bias of retrospective studies. In addition, human epidermal receptor-2 (HER-2) status was not evaluated in this study because the routine IHC test for HER-2 was not done in the investigated period. Moreover, the grade of ER positivity could not be assessed in this study. The ER grade is reported to be related with endocrine responsiveness and associated with survival outcome of ER-positive patients [12]. Because of the absence of information on HER-2 status and ER grade, further studies to define biologic effect of these factors on late recurrence still remains an unmet need. Future work with the identification of novel biomarkers as well as HER-2 and ER grade could support and strengthen our findings regarding early and late recurrence in ER-positive women.

In the article, the dissimilarity in prognostic factors between early recurrence and late recurrence in ER-positive disease was noted. Above all, this investigation highlights the diluted effect of nodal stage on late recurrence compared with early recurrence, and it suggests that tumor biology plays a more important role than tumor load in late recurrence of ER-positive disease. Our results propose that comprehensive consideration of both tumor load and tumor biology is needed to perform the translational research involving parallel comparison between early and late recurrence in this subset of patients.

## Supporting Information

Figure S1 **The goodness of fits for the binary logistic models between early recurrence within 5 years and no recurrence using Hosmer and Lemeshow test and ROC curve.**
(DOCX)

Figure S2 **The goodness of fits for the binary logistic models between late recurrence after 5 years and no recurrence using Hosmer and Lemeshow test and ROC curve.**
(DOCX)

Figure S3 **The goodness of fits for the binary logistic models between early recurrence within 5 years and late recurrence after 5 years using Hosmer and Lemeshow test and ROC curve.**
(DOCX)

Figure S4 **The goodness of fits for the binary logistic models between early metastasis within 5 years and no metastasis using Hosmer and Lemeshow test and ROC curve.**
(DOCX)

Figure S5 **The goodness of fits for the binary logistic models between late metastasis after 5 years and no metastasis using Hosmer and Lemeshow test and ROC curve.**
(DOCX)

Figure S6 **The goodness of fits for the binary logistic models between early metastasis within 5 years and late metastasis after 5 years using Hosmer and Lemeshow test and ROC curve.**
(DOCX)

Table S1 **Goodness of Fit: Hosmer and Lemeshow Test.**
(DOCX)

## References

[1] Parkin DM, Bray F, Ferlay J, Pisani P (2005) Global cancer statistics, 2002. CA Cancer J Clin 55: 74–108. 15761078 10.3322/canjclin.55.2.74

[2] Karrison TG, Ferguson DJ, Meier P (1999) Dormancy of mammary carcinoma after mastectomy. J Natl Cancer Inst 91: 80–85. 9890174 10.1093/jnci/91.1.80

[3] Peto R, Boreham J, Clarke M, Davies C, Beral V (2000) UK and USA breast cancer deaths down 25% in year 2000 at ages 20–69 years. Lancet 355: 1822. 10.1016/S0140-6736(00)02277-7 10832853

[4] Saphner T, Tormey DC, Gray R (1996) Annual hazard rates of recurrence for breast cancer after primary therapy. J Clin Oncol 14: 2738–2746. 8874335 10.1200/JCO.1996.14.10.2738

[5] Dignam JJ, Dukic V, Anderson SJ, Mamounas EP, Wickerham DL, et al (2009) Hazard of recurrence and adjuvant treatment effects over time in lymph node-negative breast cancer. Breast Cancer Res Treat 116: 595–602. 18830816 10.1007/s10549-008-0200-5 PMC2711214

[6] Hilsenbeck SG, Ravdin PM, de Moor CA, Chamness GC, Osborne CK, et al (1998) Time-dependence of hazard ratios for prognostic factors in primary breast cancer. Breast Cancer Res Treat 52: 227–237. 10066085 10.1023/a:1006133418245

[7] Early Breast Cancer Trialists’ Collaborative Group (1998) Tamoxifen for early breast cancer: an overview of the randomised trials. Lancet 351: 1451–1467. 9605801

[8] Cufer T (2007) Reducing the risk of late recurrence in hormone-responsive breast cancer. Ann Oncol 18 Suppl 8viii18–25. 17890210 10.1093/annonc/mdm262

[9] Kennecke HF, Olivotto IA, Speers C, Norris B, Chia SK, et al (2007) Late risk of relapse and mortality among postmenopausal women with estrogen responsive early breast cancer after 5 years of tamoxifen. Ann Oncol 18: 45–51. 17030545 10.1093/annonc/mdl334

[10] Carter CL, Allen C, Henson DE (1989) Relation of tumor size, lymph node status, and survival in 24,740 breast cancer cases. Cancer 63: 181–187. 2910416 10.1002/1097-0142(19890101)63:1<181::aid-cncr2820630129>3.0.co;2-h

[11] Demicheli R, Abbattista A, Miceli R, Valagussa P, Bonadonna G (1996) Time distribution of the recurrence risk for breast cancer patients undergoing mastectomy: further support about the concept of tumor dormancy. Breast Cancer Res Treat 41: 177–185. 8944336 10.1007/BF01807163

[12] Kennecke H, McArthur H, Olivotto IA, Speers C, Bajdik C, et al (2008) Risk of early recurrence among postmenopausal women with estrogen receptor-positive early breast cancer treated with adjuvant tamoxifen. Cancer 112: 1437–1444. 18286526 10.1002/cncr.23320

[13] Mansell J, Monypenny IJ, Skene AI, Abram P, Carpenter R, et al (2009) Patterns and predictors of early recurrence in postmenopausal women with estrogen receptor-positive early breast cancer. Breast Cancer Res Treat 117: 91–98. 19112615 10.1007/s10549-008-0291-z

[14] Bosco JL, Lash TL, Prout MN, Buist DS, Geiger AM, et al (2009) Breast cancer recurrence in older women five to ten years after diagnosis. Cancer Epidemiol Biomarkers Prev 18: 2979–2983. 19843686 10.1158/1055-9965.EPI-09-0607 PMC2784208

[15] Brewster AM, Hortobagyi GN, Broglio KR, Kau SW, Santa-Maria CA, et al (2008) Residual risk of breast cancer recurrence 5 years after adjuvant therapy. J Natl Cancer Inst 100: 1179–1183. 18695137 10.1093/jnci/djn233 PMC6592411

[16] Hammond ME, Hayes DF, Dowsett M, Allred DC, Hagerty KL, et al (2010) American Society of Clinical Oncology/College of American Pathologists guideline recommendations for immunohistochemical testing of estrogen and progesterone receptors in breast cancer (unabridged version). Arch Pathol Lab Med 134: e48–72. 20586616 10.5858/134.7.e48

[17] Goss P, Allan AL, Rodenhiser DI, Foster PJ, Chambers AF (2008) New clinical and experimental approaches for studying tumor dormancy: does tumor dormancy offer a therapeutic target? APMIS 116: 552–568. 18834402 10.1111/j.1600-0463.2008.001059.x

[18] Joensuu K, Hagstrom J, Leidenius M, Haglund C, Andersson LC, et al (2011) Bmi-1, c-myc, and Snail expression in primary breast cancers and their metastases–elevated Bmi-1 expression in late breast cancer relapses. Virchows Arch 459: 31–39. 21638011 10.1007/s00428-011-1096-8

[19] Joensuu K, Heikkila P, Andersson LC (2008) Tumor dormancy: elevated expression of stanniocalcins in late relapsing breast cancer. Cancer Lett 265: 76–83. 18355956 10.1016/j.canlet.2008.02.022

[20] Chen Q, Zhang XH, Massague J (2011) Macrophage binding to receptor VCAM-1 transmits survival signals in breast cancer cells that invade the lungs. Cancer Cell 20: 538–549. 22014578 10.1016/j.ccr.2011.08.025 PMC3293160

[21] Lu X, Mu E, Wei Y, Riethdorf S, Yang Q, et al (2011) VCAM-1 promotes osteolytic expansion of indolent bone micrometastasis of breast cancer by engaging alpha4beta1-positive osteoclast progenitors. Cancer Cell 20: 701–714. 22137794 10.1016/j.ccr.2011.11.002 PMC3241854

[22] Oskarsson T, Acharyya S, Zhang XH, Vanharanta S, Tavazoie SF, et al (2011) Breast cancer cells produce tenascin C as a metastatic niche component to colonize the lungs. Nat Med 17: 867–874. 21706029 10.1038/nm.2379 PMC4020577

[23] Bianchini G, Pusztai L, Iwamoto T, Kelly CM, Zambetti M, et al.. (2011) Molecular tumor characteristics influence adjuvant endocrine treatment outcome. Cancer Research 71: Supplement 3.

[24] Saghatchian M, Mittempergher L, Michiels S, Casinius S, Glas A, et al.. (2011) Characterization of breast cancer distant metastasis based on outcome over time using a gene expression profiling approach and identification of pathway activities of late relapse. Cancer Research 71: Supplement 3.

[25] Liu MC, Dixon JM, Xuan JJ, Riggins RB, Chen L, et al.. (2011) Molecular signaling distinguishes early ER positive breast cancer recurrences despite adjuvant tamoxifen. Cancer Research 71: Supplement 3.

[26] Early Breast Cancer Trialists’ Collaborative Group (2005) Effects of chemotherapy and hormonal therapy for early breast cancer on recurrence and 15-year survival: an overview of the randomised trials. Lancet 365: 1687–1717. 15894097 10.1016/S0140-6736(05)66544-0

[27] Forbes JF, Cuzick J, Buzdar A, Howell A, Tobias JS, et al (2008) Effect of anastrozole and tamoxifen as adjuvant treatment for early-stage breast cancer: 100-month analysis of the ATAC trial. Lancet Oncol 9: 45–53. 18083636 10.1016/S1470-2045(07)70385-6

