# Supplementary material for: The Difference in Prognostic Factors between Early Recurrence and Late Recurrence in Estrogen Receptor-Positive Breast Cancer: Nodal Stage Differently Impacts Early and Late Recurrence
Source: PLoS One. 2013 May 22;8(5):e63510. doi: 10.1371/journal.pone.0063510 (PMC3661516; doi:10.1371/journal.pone.0063510)
Supplement: Figure S5 — The goodness of fits for the binary logistic models between late metastasis after 5 years and no metastasis using Hosmer and Lemeshow test and ROC curve. (DOCX) [file pone.0063510.s005.docx]

**Figure S5.** The goodness of fits for the binary logistic models between late metastasis after 5 years and no metastasis using Hosmer and Lemeshow test and ROC curve

**
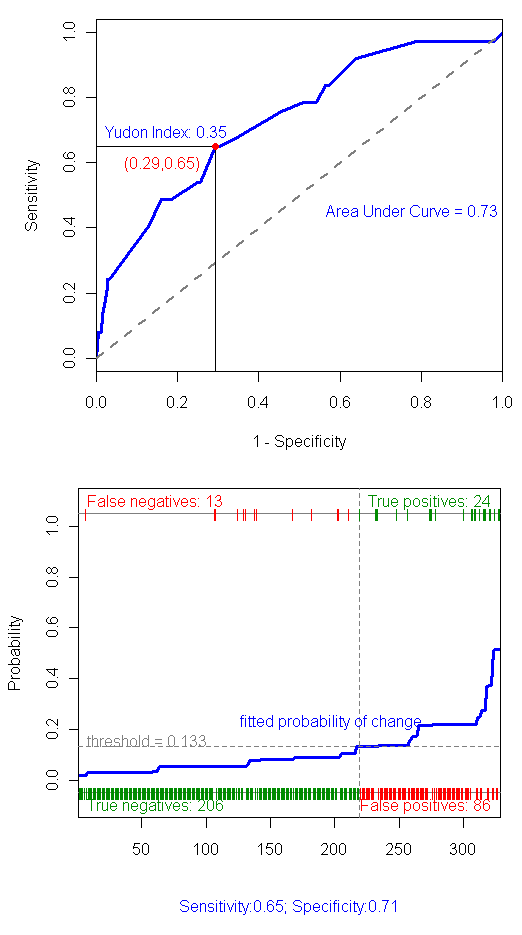
**
