# Supplementary material for: The Difference in Prognostic Factors between Early Recurrence and Late Recurrence in Estrogen Receptor-Positive Breast Cancer: Nodal Stage Differently Impacts Early and Late Recurrence
Source: PLoS One. 2013 May 22;8(5):e63510. doi: 10.1371/journal.pone.0063510 (PMC3661516; doi:10.1371/journal.pone.0063510)

**Figure S6.** The goodness of fits for the binary logistic models between early metastasis within 5 years and late metastasis after 5 years using Hosmer and Lemeshow test and ROC curve


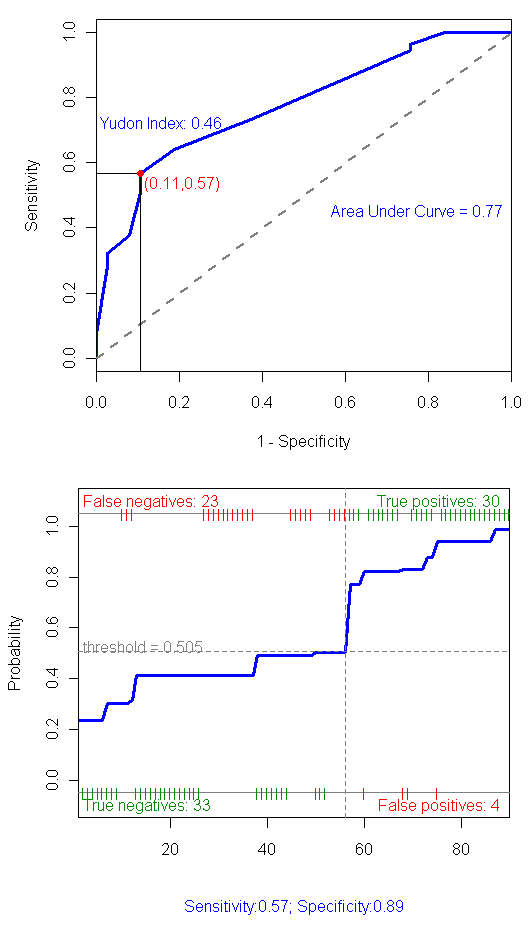

Supplement: Figure S6 — The goodness of fits for the binary logistic models between early metastasis within 5 years and late metastasis after 5 years using Hosmer and Lemeshow test and ROC curve. (DOCX) [file pone.0063510.s006.docx]
