# Supplementary material for: The Difference in Prognostic Factors between Early Recurrence and Late Recurrence in Estrogen Receptor-Positive Breast Cancer: Nodal Stage Differently Impacts Early and Late Recurrence
Source: PLoS One. 2013 May 22;8(5):e63510. doi: 10.1371/journal.pone.0063510 (PMC3661516; doi:10.1371/journal.pone.0063510)
Supplement: Table S1 — Goodness of Fit: Hosmer and Lemeshow Test. (DOCX) [file pone.0063510.s007.docx]

**Table S1.** Goodness of Fit: Hosmer and Lemeshow Test

| Model | chi-square | df | *p* value |
| --- | --- | --- | --- |
| Early recur<5 & Late recur>5 | 3.378 | 6 | 0.760 |
| Early recur<5 & No recur | 8.961 | 7 | 0.255 |
| Late recur>5 & No recur | 4.670 | 6 | 0.587 |
| Early SR recur<5 & Late SR recur>5 | 4.639 | 6 | 0.591 |
| Early SR recur<5 & No SR recur | 14.545 | 8 | 0.069 |
| Late SR recur>5 & No SR recur | 9.193 | 8 | 0.326 |
